# Supplementary material for: Phactr4 influences macrophage lamellipodial structure and dynamics through Arp2/3 complex and Ezrin regulation
Source: bioRxiv. 2025 May 14:2025.05.13.653717. Preprint. [Version 1] doi: 10.1101/2025.05.13.653717 (PMC12132572; doi:10.1101/2025.05.13.653717)

bioRxiv preprint doi: https://doi.org/10.1101/2025.05.13.653717; this version posted May 14, 2025. The copyright holder for this preprint (which was not certified by peer review) is the author/funder. This article is a US Government work. It is not subject to copyright under 17 USC 105 and is also made available for use under a CC0 license.

Supplemental Figure 1. Phactr4 deletion in macrophages leads to a cell spreading defect following serum depletion and re-addition. (A) Representative differential interference contrast (DIC) images of siControl and siPhactr4 BMDMs. Cells were plated on 10 µg/mL fibronectin and allowed to spread overnight prior to imaging. Scale bar = 10 µm. (B) Representative immunofluorescence images of F-actin (gray) in siControl and siPhactr4 immortalized macrophages. Cells were either left untreated (left), subjected to serum-free media

bioRxiv preprint doi: https://doi.org/10.1101/2025.05.13.653717; this version posted May 14, 2025. The copyright holder for this preprint (which was not certified by peer review) is the author/funder. This article is a US Government work. It is not subject to copyright under 17 USC 105 and is also made available for use under a CC0 license.

for 2 hours (middle), or serum-starved for 2 hours followed by 2 hours of serum re-addition (right) prior to fixation and staining. Scale bar = 20 μm. (C) Morphological analysis of cell area (μm²), perimeter (μm), and sphericity (arbitrary units). Automated analysis was performed using Olympus cellSens image analysis software, which masked individual cells based on F-actin fluorescence. Approximately 100 cells per condition, per experiment were analyzed, with N = 3 independent experiments. Statistical analysis was performed using Kruskal-Wallis with Dunn's multiple comparisons test. Area (NT) siControl vs. siPhactr4 **p = 0.0091. Area (Serum Re-Addition) siControl vs. siPhactr4 **p = 0.0011.

Supplemental Figure 2. Phactr4-deficient macrophages undergo complement-mediated phagocytosis at a low rate, but demonstrate phagolysosome maturation comparable to siCon. (A) Representative differential interference contrast (DIC) images merged with the DsRed fluorescence channel of individual siControl (top) and siPhactr4 (bottom) macrophages at 1-hour, 3-hour, and 6-hour time intervals. The DsRed signal becomes fluorescent as the pHrodo-opsonized bead is internalized and enters the phagolysosome. (B) Quantification of phagolysosome area and mean fluorescence intensity (MFI) of the fluorescent signal. Phagolysosome area was quantified using Olympus CellSens image analysis software to mask the fluorescent signal at each time point of interest. The total area of each phagolysosome and the fluorescence MFI were averaged for each experiment. Data represent N = 3 independent experiments, with each experiment consisting of 10–15 fields of view and approximately 10–20 cells per frame. Statistical analysis was performed using Kruskal-Wallis with Dunn's multiple comparisons test, with all siControl versus siPhactr4 comparisons not significant.

Supplemental Figure 3. Additional kymography data for siCon and siPh4 macrophages. (A) Representative differential interference contrast (DIC) images showing a close-up of the leading edge of siControl and siPhactr4 macrophages at 100× magnification. Cells are BMDMs that underwent double siRNA transfection 7 days after isolation from bone marrow. siRNA knockdown was validated prior to imaging. Cells were plated on 10 μg/mL fibronectin and imaged 1 day after plating. Each color represents the cell edge outline at 1-minute intervals over a 5-minute period. White lines indicate 2–3 kymography lines drawn per leading edge in regions displaying visually dynamic behavior. Scale bar = 5 μm. (B) Quantification of displacement in μm of protrusion and retraction segments of siCon and siPh4 kymographs. (C) Example kymographs of siControl and siPhactr4 macrophage cell edge dynamics, highlighting stalled membrane segments. Stalled regions are indicated by white lines and red arrows.

bioRxiv preprint doi: https://doi.org/10.1101/2025.05.13.653717; this version posted May 14, 2025. The copyright holder for this preprint (which was not certified by peer review) is the author/funder. This article is a US Government work. It is not subject to copyright under 17 USC 105 and is also made available for use under a CC0 license.

Supplemental Figure 4. Additional phosphorylation events in siPh4 cells, rescue of siPh4 phenotype with ezrin inhibition, and supporting data on Arp2/3 complex-Phactr4 interaction. (A) Western blot analysis of (L to R) phosphorylated myosin light chain (pMLC, Ser20), phosphorylated cofilin (pCofilin, Ser3), vinculin, and phosphorylated Arp2 (pArp2, Thr-237/T-238). GAPDH was used as a loading control and for blot normalization. N = 8 for pMLC, N = 4 for pCofilin and vinculin, and N = 6 for pArp2, all independent paired siCon and siPh4 lysates. (B) Representative confocal images of proximity ligation assay (PLA) signal from the Duolink PLA assay, shown as maximum projections from a z-stack. Fluorescent puncta represent individual localizations of Ezrin-Phactr4 interactions. Cells were either untreated (left), treated with 100 μM CK-666 for 2 hours (middle), or treated with 100 μM CK-666 for 2 hours followed by a 10-minute media washout (right) before fixation and PLA assay. As signal in the washout samples was routinely low, a line was drawn around example cells in this image to denote cell volume based on companion relief contrast image. Scale bar = 10 μm for all images. Quantification of PLA fold change (right). PLA signal was normalized to the no-treatment (NT) control. Means and standard error of the mean are represented. (C) Additional kymography analysis quantification of protrusion duration and retraction duration. Statistical analysis was performed using Welch's t-test. *p = 0.0444 (Retraction duration: siControl vs. siPhactr4 No Treatment).

Supplemental Figure 5. Supporting data on Arp2/3 complex-Phactr4 interaction. (A) Representative images of WT macrophages under control conditions (top) or induced spreading following CK-666 treatment and an 8-minute media washout (bottom). Scale bar = 10 μm. (B) Quantification of the percentage of the cell edge with localized Phactr4. Means and standard error of the mean are represented by black symbols, with each experiment's mean indicated. All data points are plotted, and each experimental run is color-coded with corresponding symbols. Each experiment includes 15 cells per condition, with N = 3 independent experiments. Statistical analysis was performed using Welsh's t-test, ****p < 0.0001.

LIST OF ACCOMPANYING SUPPLEMENTARY MOVIES

Supplemental Movie 1. Time-lapse imaging of siControl (left) and siPhactr4 (right) macrophages during a random migration assay. Scale bar = 50 microns

bioRxiv preprint doi: https://doi.org/10.1101/2025.05.13.653717; this version posted May 14, 2025. The copyright holder for this preprint (which was not certified by peer review) is the author/funder. This article is a US Government work. It is not subject to copyright under 17 USC 105 and is also made available for use under a CC0 license.

Supplemental Movie 2. Time-lapse imaging of siControl (left) and siPhactr4 (right) macrophages during a phagocytosis assay. Scale bar = 50 microns

Supplemental Movie 3. 100x DIC imaging of siControl (left) and siPhactr4 (right) leading edges used in kymography analysis. Scale bar = 10 microns

Supplemental Movie 4. Time-lapse imaging of WT fibroblasts expressing GFP-Control (left) and GFP-Phactr4 (right). Scale bar = 10 microns

Supplemental Movie 5. 100x DIC imaging of vehicle-treated (left) and Ezrin inhibitor-treated (right) siPh4 cell leading edges used in kymography analysis. Scale bar = 10 microns

Supplemental Movie 6. Time-lapse imaging of WT fibroblasts expressing GFP-Phactr4 treated with DMSO (left) or CK-666 (right). Scale bar = 10 microns

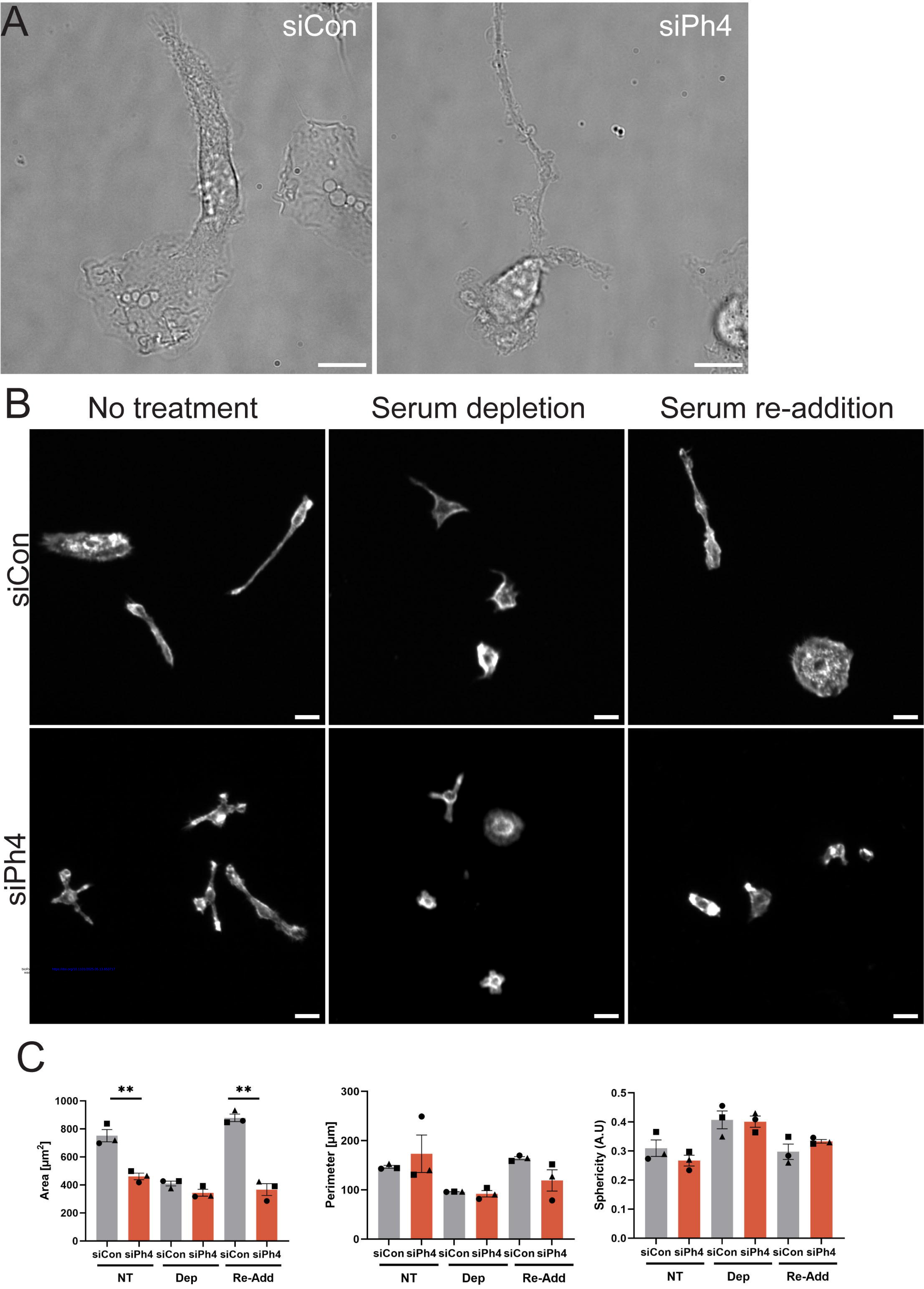
A
siCon
siPh4
B
No treatment
Serum depletion
Serum re-addition
siCon
siPh4
C
Area [µm²]
1000
800
600
400
200
0
**
**
Perimeter [µm]
300
200
100
0
Sphericity (A.U)
0.5
0.4
0.3
0.2
0.1
0.0
siCon siPh4 siCon siPh4 siCon siPh4
NT
Dep
Re-Add

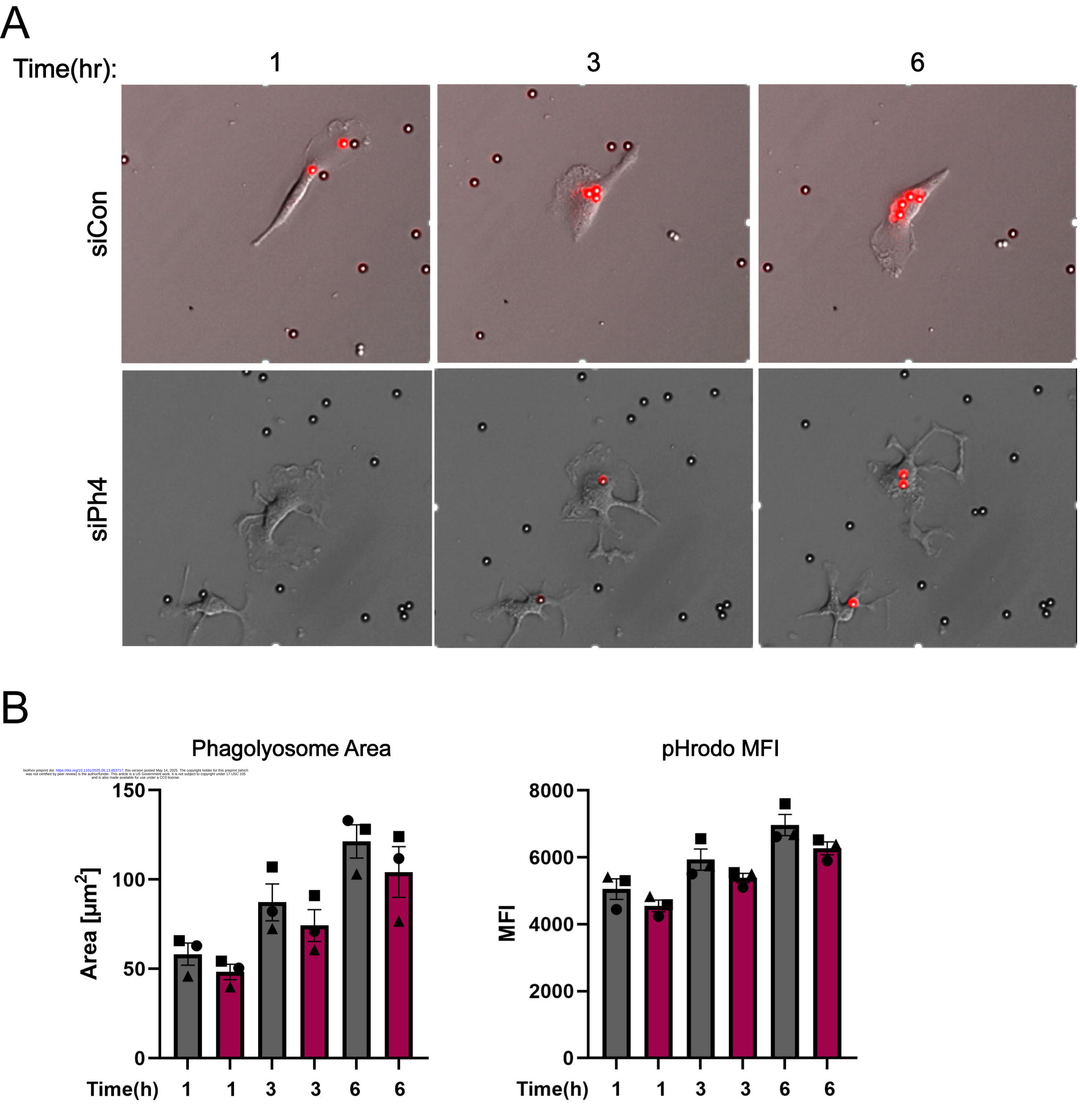

bioRxiv preprint doi: https://doi.org/10.1101/2025.05.13.653717; this version posted May 14, 2025. The copyright holder for this preprint (which was not certified by peer review) is the author/funder. This article is a US Government work. It is not subject to copyright under 17 USC 105 and is also made available for use under a CC0 license.

bioRxiv preprint doi: https://doi.org/10.1101/2025.05.13.653717; this version posted May 14, 2025. The copyright holder for this preprint (which was not certified by peer review) is the author/funder. This article is a US Government work. It is not subject to copyright under 17 USC 105 and is also made available for use under a CC0 license.

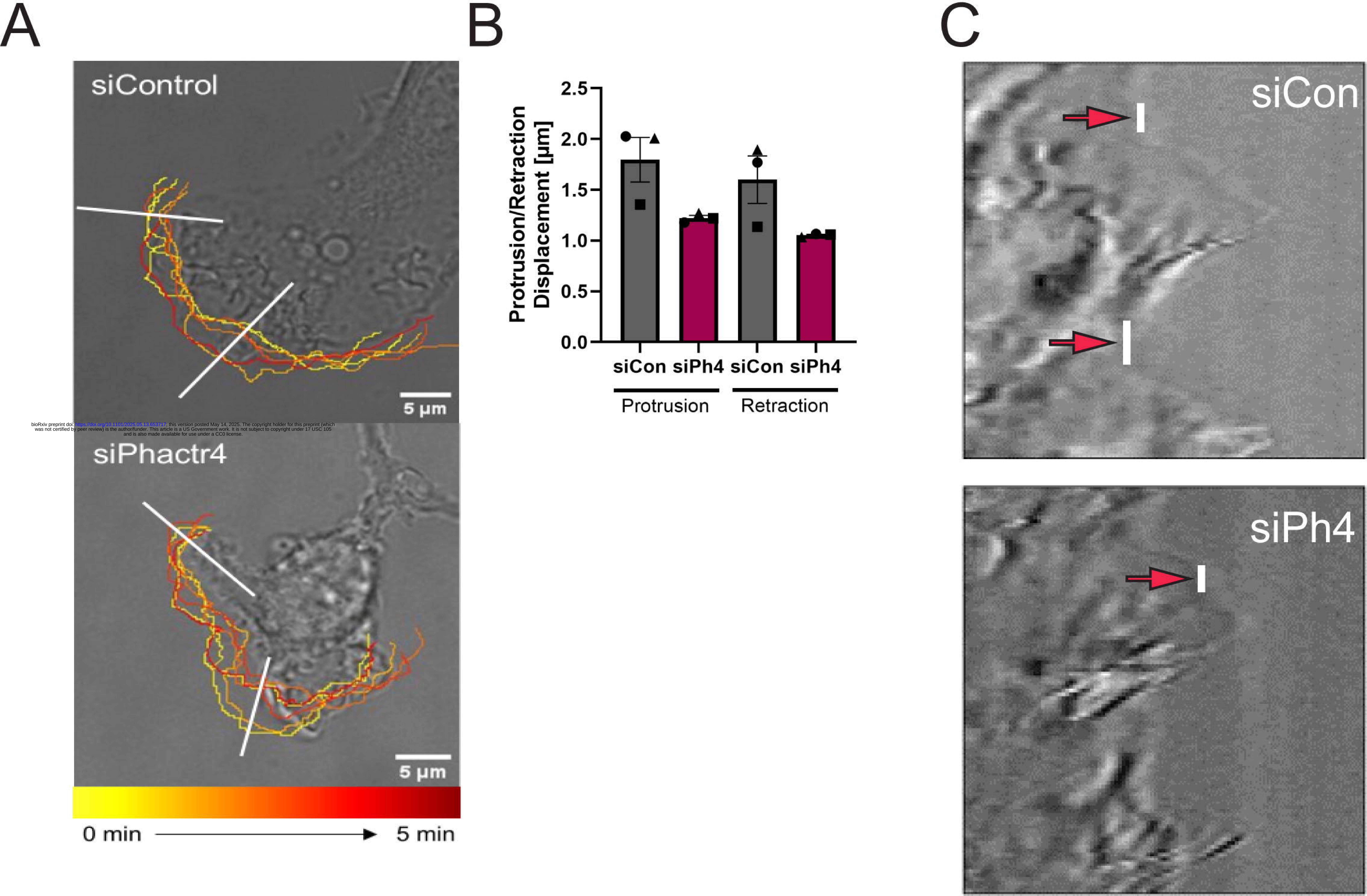

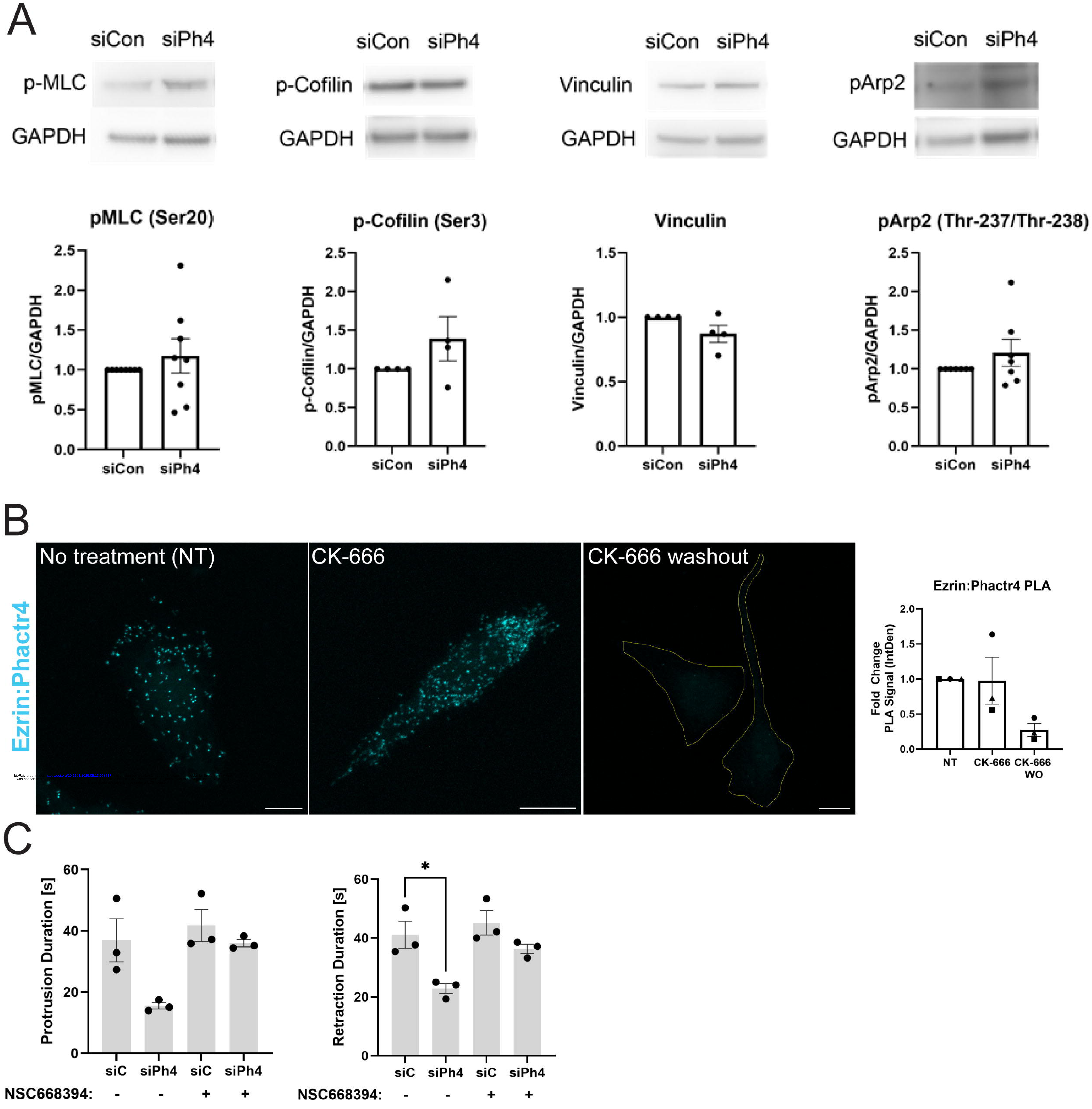
A
siCon
siPh4
p-MLC
GAPDH
p-Cofilin
Vinculin
pArp2
pMLC (Ser20)
pMLC/GAPDH
p-Cofilin (Ser3)
p-Cofilin/GAPDH
Vinculin/GAPDH
pArp2 (Thr-237/Thr-238)
pArp2/GAPDH
B
No treatment (NT)
CK-666
CK-666 washout
Ezrin:Phactr4
Ezrin:Phactr4 PLA
Fold Change
PLA Signal (IntDen)
NT
CK-666
CK-666 WO
C
Protrusion Duration [s]
Retraction Duration [s]
siC
siPh4
NSC668394:

bi[illegible]print [illegible] https://doi.org/10.1101/2025.05.13.653717
was not certifie[illegible] under a CC0 license.

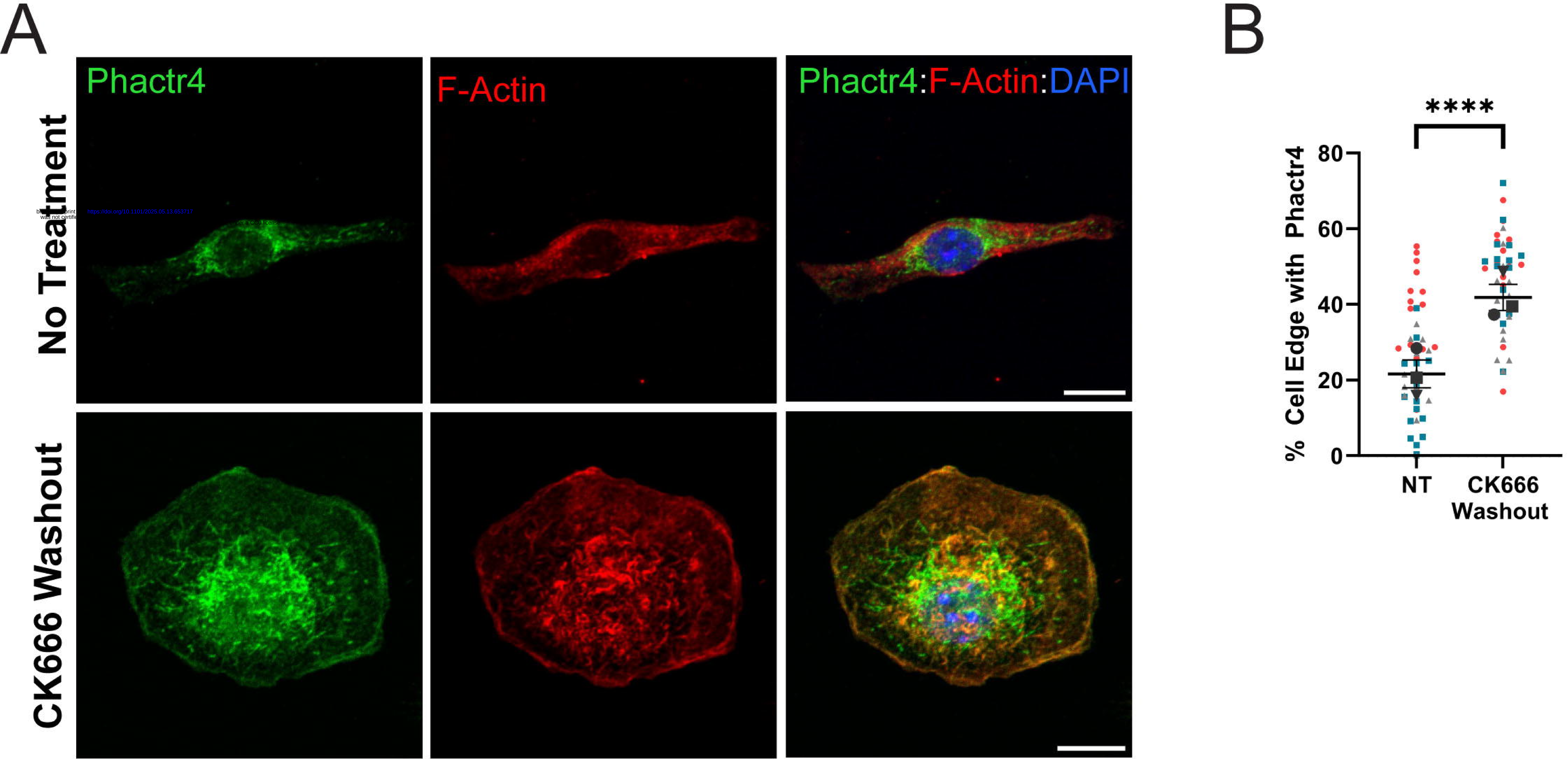

Supplement: Supplement 7 [file NIHPP2025.05.13.653717v1-supplement-7.pdf]
